# Climate Change and the Potential Distribution of an Invasive Shrub, *Lantana camara* L

**DOI:** 10.1371/journal.pone.0035565

**Published:** 2012-04-19

**Authors:** Subhashni Taylor, Lalit Kumar, Nick Reid, Darren J. Kriticos

**Affiliations:** 1 Ecosystem Management, School of Environmental and Rural Science, University of New England, Armidale, New South Wales, Australia; 2 CSIRO Ecosystem Sciences, Canberra, Australian Capital Territory, Australia; 3 E. H. Graham Centre, Charles Sturt University, Wagga Wagga, New South Wales, Australia; Pacific Climate Impacts Consortium, Canada

## Abstract

The threat posed by invasive species, in particular weeds, to biodiversity may be exacerbated by climate change. *Lantana camara* L. (lantana) is a woody shrub that is highly invasive in many countries of the world. It has a profound economic and environmental impact worldwide, including Australia. Knowledge of the likely potential distribution of this invasive species under current and future climate will be useful in planning better strategies to manage the invasion. A process-oriented niche model of *L. camara* was developed using CLIMEX to estimate its potential distribution under current and future climate scenarios. The model was calibrated using data from several knowledge domains, including phenological observations and geographic distribution records. The potential distribution of lantana under historical climate exceeded the current distribution in some areas of the world, notably Africa and Asia. Under future scenarios, the climatically suitable areas for *L. camara* globally were projected to contract. However, some areas were identified in North Africa, Europe and Australia that may become climatically suitable under future climates. In South Africa and China, its potential distribution could expand further inland. These results can inform strategic planning by biosecurity agencies, identifying areas to target for eradication or containment. Distribution maps of risk of potential invasion can be useful tools in public awareness campaigns, especially in countries that have been identified as becoming climatically suitable for *L. camara* under the future climate scenarios.

## Introduction

Biotic invasions occur when organisms are transported to new areas where they may reproduce and spread. Such invasions can have far reaching ecological and economic impacts [1]–[4]. Along with climate, such invasions are now seen as a contributor to global change [3]. Invasive species are a major threat to the Earth's biodiversity because they often dramatically affect the structure and functioning of ecosystems [5]. Prudent management of biological invasions requires information about the expected potential distribution and relative abundance of invasive species under current and future climate scenarios. Such information is necessary for risk assessment as well as the formulation of appropriate long-term management strategies. Species distribution models (SDMs), bioclimatic models, and ecological niche models (ENMs) [6] provide many opportunities in this area. Niche modelling is underpinned by Hutchinson's [7] fundamental and realized niche concepts. However, there are conflicting views on what the models actually represent [8]. While some researchers suggest that niche models provide an estimate to the species' fundamental niche [9], others consider models as presenting a “spatial representation of the realized niche" [6], [10]. The distribution of a species depends on complex interactions between a range of factors, acting with different strengths at different scales [10]. These include abiotic and biotic factors, the foremost of which is climate [11]–[12].

CLIMEX [13] is a useful tool for exploring the relationship between the fundamental and realized niche of species [14]. It is an eco-climatic modelling package that has been used by many researchers involved in estimating invasive species' potential distributions under current and future climate [15]–[18]. The realized niche of a species is the range of conditions and resources in which it can persist in the presence of competitors and predators [19] and this is represented by the native range of a species [20]. After introduction into an exotic environment, a species can commonly inhabit a broader range of climatic conditions because it is freed from many of its competitors and predators. This is potentially its fundamental niche [7]. It is important to include exotic range data when developing climate models for invasive species [21] because any predictions based on just its native range may lead to an underestimation of the species' potential range, especially if it has not had the opportunity to express its full climatic preferences in the absence of natural enemies [20].

CLIMEX allows users to model the potential distribution of organisms, drawing upon a variety of information types, including direct experimental observations of a species' growth response to temperature and soil moisture, its phenology and knowledge of its current distribution. In a review of the various climate-based packages designed to estimate potential species distributions, Kriticos and Randall [21] found that ‘CLIMEX was the most suitable climate modelling package for undertaking Weed Risk Assessments because it can support model-fitting to a global plant distribution, includes a climate change scenario mechanism, and provides an insight into the plant's ecological response to climate’. Subsequently, Webber et al. [22] found that CLIMEX was better placed than two correlative modelling methods (MaxEnt and Boosted Regression Trees) to project a species' distribution in a novel climate such as a new continent, or under a future climate scenario. Modelling the potential distribution of a species using climatic mapping has received some criticism because it assumes that climate alone limits the geographical distribution and does not include biotic interactions and dispersal [23]. However, despite its limitations, climatic mapping plays an important role in the definition of the fundamental (potential) niche of an invasive species in its exotic range [24]. Since climate is one of the major determinants of the potential range of species, climate changes could have a significant impact on species' distributions.

There is now overwhelming evidence for rapid climate change with global mean surface temperatures projected to increase by 2.4 to 6.4°C between 1990 and 2100 [25] along with various changes in rainfall patterns (increases, decreases and changes in seasonality). Together with major threats to biodiversity, agriculture and human health, climate change also has implications for invasive species. The immediate effect of climate change on such species will most likely be shifts in their distributions facilitated by changes in temperature and rainfall patterns that define their range boundaries. Species that can tolerate a wide range of climatic conditions may be favoured, and as a result they may have greater competitive success than most native species [26]–[28].

One such species, *Lantana camara* L. (lantana), is a major weed in many tropical and subtropical countries outside its native range of Central and northern South America and the Caribbean. Its global distribution includes approximately 60 countries or island groups between 35°N and 35°S [1]. It has a variety of impacts including a reduction in native species diversity, extinctions, decline in soil fertility, and allelopathic alteration of soil properties as well as an alteration of ecosystem processes [1]. Allelochemicals found in lantana have been shown to inhibit the growth of other species growing close to it [29]–[30]. Persistent lantana infestations can lead to a reduction in biodiversity because it has the potential to block succession and cause the displacement of native species [31]–[32]. It can cause striking changes in the structural and floristic composition of natural communities by interrupting the regeneration processes of other native species thus reducing species richness [33].

This study utilized the CLIMEX modelling package to develop a model of the climate responses of lantana based on its native distribution and invasive distribution outside Australia. This model was then used to project its potential distribution under current climate, using the extensive Australian distribution data for model validation and assess the impacts of climate change on its potential distribution using two global climate models (GCM), CSIRO-Mk3.0 and MIROC-H. These were run with the A1B and A2 SRES (Special Report on Emissions Scenarios) emission scenarios for 2030 and 2070.

## Materials and Methods

### CLIMEX Software

CLIMEX for Windows Version 3 [13], [34]–[35] was used to develop a model of the potential distribution of *L. camara* under current and future climate scenarios. CLIMEX is based on the observation that the distribution of plants and poikilothermal animals is primarily determined by climate [36]. The software works on the basis of an eco-physiological model that assumes that at each location, a species may experience a favourable season with positive population growth and an unfavourable season that causes population decline [35]. The user can use the model to infer parameters that describe the species' response to climate based on its geographic range or phenological observations [35]. CLIMEX can also be used deductively to apply climate response parameters extracted from experimental observations to climatic datasets. In practice, both approaches can be applied to inform the selection of parameter values. These parameters can then be applied to novel climates to project the species' potential range in new regions or climate scenarios [22], [37]. The potential for population growth during favourable climate conditions is described by an annual growth index (GI_A_) that conforms to the law of tolerance [38] and the law of minimum [39]. Four stress indices (cold, wet, hot and dry) and up to four interaction stresses (hot–dry, hot–wet, cold–dry and cold–wet) are used to describe the probability that the population can survive unfavourable conditions. The growth and stress indices are calculated weekly and combined into an overall annual index of climatic suitability, the Ecoclimatic index (EI) which is theoretically scaled from 0 to 100. Establishment is only possible if EI>0. In practice, EI values close to the maximum are rare, and confined to species with an equatorial range, as this would imply ideal growing conditions year-round [40]. EI values close to zero indicate a low probability of conditions conducive to persistence in time and space. In such marginally suitable climates, species are likely to be restricted to favourable microhabitats, and to exhibit significant metapopulation dynamics.

### Taxonomy and Native and Naturalized Distribution of *L. camara*

The genus *Lantana* L. (Verbenaceae) includes up to 150 species [41]–[42]. Many of these species are native to South America, Central America or southern North America, while a few species occur naturally in Africa and Asia [43]. There is considerable uncertainty associated with the taxonomy of the genus *Lantana*. Four distinct groups can be recognized within the genus [44]. These are referred to as *Lantana* sections *Calliorheas*, *Sarcolippia*, *Rhytocamara* and *Camara*. *Lantana* section *Camara* is divided into three complexes based on *L. urticifolia*, *L. hirsuta* and *L. camara*. The *L. camara* complex contains the weedy lantana generally referred to as *L. camara* L. *sensu lato*, which has a pan-tropical distribution [1]. *Lantana camara sensu stricto* is known from Jamaica, Trinidad, Mexico, Brazil and Florida [44]. It may have a wider native distribution in South America [45] extending to Argentina and Uruguay [1], [46]. The present study only addresses the ‘weedy taxa’ of *Lantana* section *Camara* which are the most prevalent taxa in the genus. They are important due to economic and environmental impacts as they can invade natural and agricultural ecosystems [47]–[48]. Its environmental impacts are especially damaging in native forests that have undergone disturbance. In such cases, lantana forms a dense understorey, disrupts succession and decreases biodiversity [1], [32]. In areas that have a high density of lantana, species richness is reduced [33], [49] and local flora is threatened [50]–[51]. In natural systems, dense lantana infestations can alter fire regimes [52]. Lantana is a weed of important crops such as coffee, oil palms, coconuts, cotton, bananas, pineapples, sugarcane, tea, rubber and rice in various countries [53]. It forms dense thickets in pastures, outcompeting desirable pasture species and rendering infested areas useless for pasture [1], [53]. Within the ‘weedy taxa’, there are many variants of *L. camara*, referred to here as varieties. Twenty-nine varieties are recognized in Australia [54]. The common name lantana is used in the remainder of the paper to refer to the weedy taxa of the section *Camara*.

**Figure 1:**
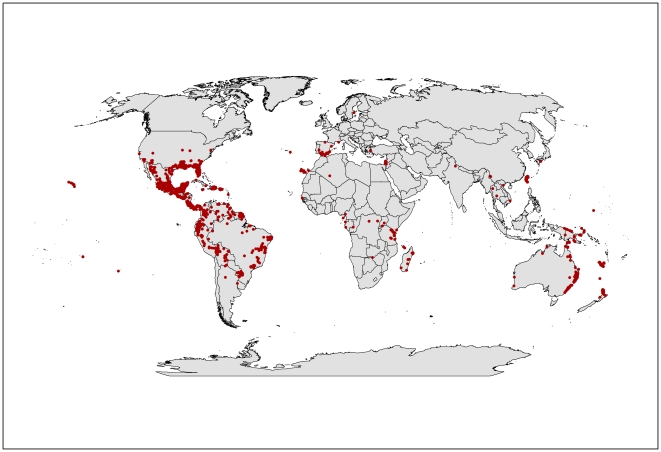
The current global distribution of *L. camara* taken from the Global Biodiversity Information Facility 2007. Red dots indicate occurrence records of *L. camara*.

The Global Biodiversity Information Facility (GBIF) is a database of natural history collections around the world for various species and is available for download. Information on *L. camara* distribution was downloaded [55] (Figure 1) and used in parameter fitting. Some 4126 records were downloaded but many did not have geographic coordinates and were removed, leaving 2753 records. However, many of these records were duplicates and were also removed. Thus 1740 records from the GBIF database were used in parameter fitting. Distribution data from South Africa [56] and Asia [57]–[61] were also obtained to assist in fitting parameters. Seasonal phenology data for the southern states of Brazil were used to fit growth parameters [62], [63]. Although Winder's seasonal phenology observations were restricted to *Lantana tiliaefolia* and *L. glutinosa*, the ecology of these two species are similar to the weedy taxa of lantana, and thus these data were used in parameter fitting.

### Climate Data and Climate Change Scenarios

The CliMond 10′ gridded climate data [64] were used for modelling. Average minimum monthly temperature (*T*_min_), average maximum monthly temperature (*T*_max_), average monthly precipitation (*P*_total_) and relative humidity at 09:00 h (*RH*_09:00_) and 15:00 h (*RH*_15:00_) were used to represent historical climate (averaging period 1950–2000). The same five variables were used to characterize potential future climate in 2030 and 2070, based on two Global Climate Models (GCMs), CSIRO-Mk3.0 [65] and MIROC-H (Centre for Climate Research, Japan) with the A1B and A2 SRES scenarios [66]. These were available as part of the CliMond dataset. The two GCMs were selected from 23 GCMs for the CliMond dataset based on three criteria [64]:

1. The temperature, precipitation, mean sea level pressure and specific humidity variables required for CLIMEX were available for these two GCMs.
2. The models have relatively small horizontal grid spacing.
3. They performed well compared to other GCMs in representing basic aspects of observed climate at a regional scale [67].

The A1B and A2 scenarios were selected to typify the range of possible climate suitability for *L. camara* in 2030 and 2070. No scenarios from the B family of SRES scenarios were included in this paper because recent analyses of trends in factors such as global temperature and sea rise [68] showed that the observed increases were much higher than the hottest SRES scenario. The A1B scenario describes a balance between the use of fossil and non-fossil resources while A2 describes a varied world with high population growth but slow economic development and technological change. The projection dates of 2030 and 2070 were chosen because they provide a reasonable snapshot of two periods, one in the near future in 20 years' time and one much later in the future in 60 years' time.

### Fitting CLIMEX Parameters

Sutherst [40] and Kriticos and Leriche [69] suggested that using both native and exotic distribution data in fitting CLIMEX parameters could produce a model that better approximates the potential distribution of the taxa being modelled than one that relies solely on native range data. They suggested that the constraints imposed by biotic influences in the species' native range may be absent in exotic locations, thus allowing it to expand its range beyond its Hutchinsonian realized niche [70]. Stress parameters were fitted to the known native (Central and South America) and naturalized (South Africa and Asia) distribution of the species while the phenology data from Brazil was used to fit growth parameters [62]–[63]. Each of the parameters was adjusted iteratively until a satisfactory agreement was reached between the potential and known distribution of lantana in these areas. The fitted parameters were checked to ensure that they were biologically reasonable. Australian distribution data was reserved for validation of the model.

### Cold Stress

Two cold stress mechanisms were used to define the southern limits of lantana distribution in Argentina and northern limits in Nepal, Pakistan and China. Lantana seldom occurs where temperatures frequently fall below 5°C [71], and prolonged freezing temperatures kill aerial woody branches and cause defoliation [1]. Therefore, intolerance to frost was incorporated by accumulating stress when the average monthly minimum temperature fell below 5°C with the frost stress accumulation rate (THCS) set at −0.004 week^−1^. This cold-stress mechanism allowed the species to survive in Kathmandu (27°42′N 85°18′E) [72]. The Cold-Stress Degree-day Threshold (DTCS) was set at 15°C days, with the stress accumulation rate (DHCS) set at −0.0022 week^−1^ so that the potential distribution was restricted to the known southern limits in Buenos Aires and northern limits in India, Nepal and China. This form of cold stress accounts for the need for the plant to grow at a minimal rate in order to offset respiration losses. If the temperatures are insufficient for the plant to grow this minimal amount, it needs to draw on photosynthate reserves.

### Heat Stress

The heat stress parameter (TTHS) was set at 33°C, the same level as the limiting high temperature (DV3) with a stress accumulation rate (THHS) of 0.001 week^−1^, which allowed lantana to persist along the Western Ghats [73] as well as in Bengal and Assam in India where it is reportedly common [57].

### Dry Stress

The dry stress parameter was set at the same level (0.1) as the lower soil moisture threshold (SM0) because soil moisture related stresses probably begin at the same soil moisture levels where growth stops. The stress accumulation rate of −0.01 week^−1^ was set to exclude the species from the drier western parts of South Africa where it survives only as an ornamental plant [74].

### Wet Stress

The wet stress threshold (SMWS) was set to 1.6 and the accumulation rate (HWS) set at 0.01 week^−1^ since lantana can tolerate up to 3000 mm of rainfall per year as long as the soil is not waterlogged for prolonged periods [1], [75]. These settings allowed the species to grow well in Indonesia and the Philippines [53] as well as in central Burma, but excluded it from the wetter coastal areas [57].

### Temperature Index

In South Africa, lantana is found in areas with a mean annual surface temperature greater than 12.5°C [76]. The seasonal phenology data for Iguazu (25°33′S, 54°34′W) in Brazil showed that ‘cold winter temperatures caused cessation of growth with a substantial loss in leaves and side-branches’ [63]. Winter temperatures in Iguazu can get as low as 8°C. Thus, the limiting low temperature (DV0) was set at 10°C to reduce growth appropriately during winter months in Iguazu. This value was chosen as a compromise between the South African distribution data and the phenology data from Iguazu. According to Day et al. [1], lantana does not appear to have an upper temperature limit. The summer temperatures in Iguazu rarely exceed 33°C and thus the limiting high temperature DV3 was set at 33°C, which allowed it to survive in Iguazu where it grows rapidly during summer [63]. The lower (DV1) and upper (DV2) optimal temperatures were set at 25°C and 30°C, respectively, based on seasonal phenology at Iguazu, and these provided a good fit to the observed distribution in South America, Asia and South Africa.

### Moisture Index

The lower moisture threshold (SM0) was set at 0.1, corresponding to the permanent wilting point for many plants [35]. This excluded lantana from the drier western parts of South Africa where it survives only as an ornamental [74] but allowed it to survive in Israel where Danin [77] reported lantana as ‘a common component of the wasteland vegetation in the lowlands of the Mediterranean territories of Israel’. However, lantana may survive in certain areas of Israel due to irrigation since one of its other common habitats is irrigated cultivation such as date palm plantations and orchards [77]. The lower (SM1) and upper (SM2) optimum moisture thresholds were set at 0.5 and 1.2, respectively, to improve species growth during the months of January to March in Iguazu [63]. The upper soil moisture threshold (SM3) was set at 1.6 to allow it to grow in the Philippines and Indonesia where it has been reported as a troublesome weed [53].

### Annual Heat Sum

The PDD thermal accumulation (number of degree days) mechanism did not appear to contribute to the definition of the South American or Asian distribution and so this parameter was not used.

The parameters are shown in Table 1. These parameters were used to model potential lantana distribution under the reference climate (averaging period 1950–2000) as well as climate change scenarios described above.

**Table 1:** The CLIMEX parameter values that were used for *L. camara*.

Parameter	Values
Limiting low temperature (DV0)	10°C
Lower optimal temperature (DV1)	25°C
Upper optimal temperature (DV2)	30°C
Limiting high temperature (DV3)	33°C
Limiting low soil moisture (SM0)	0.1
Lower optimal soil moisture (SM1)	0.5
Upper optimal soil moisture (SM2)	1.2
Limiting high soil moisture (SM3)	1.6
Cold stress temperature threshold (TTCS)	5°C
Cold stress temperature rate (THCS)	−0.004 week^−1^
Minimum degree-day cold stress threshold (DTCS)	15°C days
Degree-day cold stress rate (DHCS)	−0.0022 week^−1^
Heat stress temperature threshold(TTHS)	33°C
Heat stress temperature rate (THHS)	0.001 week^−1^
Dry stress threshold (SMDS)	0.1
Dry stress rate (HDS)	−0.01 week^−1^
Wet stress threshold (SMWS)	1.6
Wet stress rate (HWS)	0.01 week^−1^

## Results

### Current Climate

**Figure 2:**
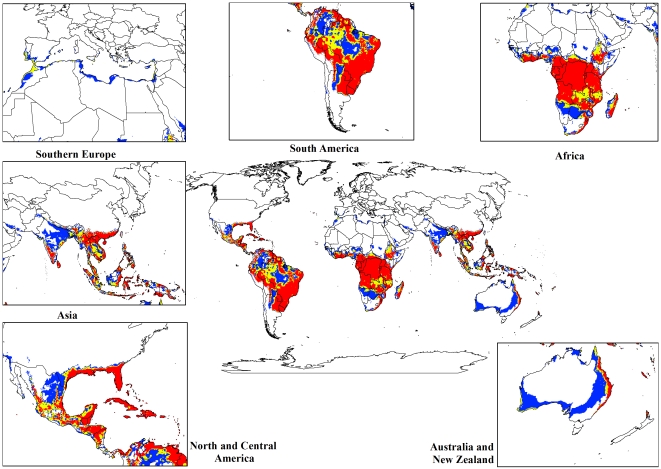
The climate (EI) for *L. camara* based on CLIMEX for reference climate (averaging period 1950–2000). White areas indicate unsuitable climate areas (EI = 0), blue areas indicate marginal climate areas (EI = 1–10), yellow areas indicate suitable climate areas (EI = 10–20) and red areas indicate highly suitable climate areas (EI>20).

The modelled global climate suitability for lantana (Figure 2) compares well with its known native distribution in South and Central America as well as its exotic range in South Africa and Asia (Figure 1). A comparison of Figures 1 and 2 showed that the present global distribution of lantana is consistent with the Ecoclimatic Index values resulting from the CLIMEX model. Much of the tropics and subtropics are projected to have suitable climatic conditions for lantana. Large areas of South and Central America, the southern states of the USA, Asia, sub-Saharan Africa, Madagascar and the high volcanic Pacific island groups such as Fiji, Vanuatu, Samoa and New Caledonia, among others, have highly suitable climate for the species. Warm temperate areas such as northern New Zealand and southern Mediterranean Europe including Portugal, Italy and Greece are predicted to have unsuitable climates.

**Figure 3:**
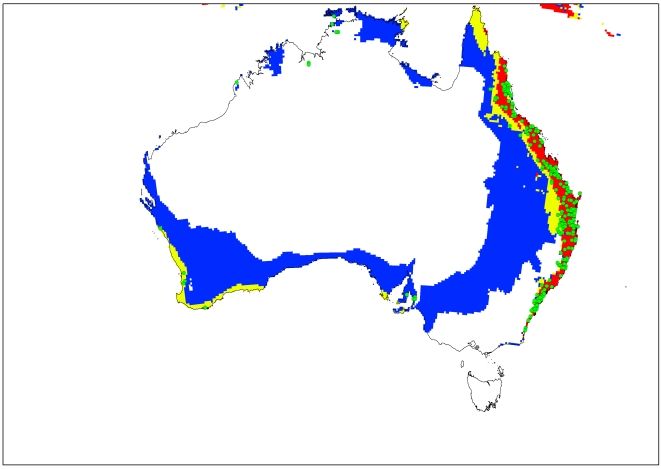
Current and modelled climate (EI) for *L. camara* based on CLIMEX for reference climate (averaging period 1950–2000). Data for current Australian distribution is taken from Australia's Virtual Herbarium. White areas indicate unsuitable climate areas (EI = 0), blue areas indicate marginal climate areas (EI = 1–10), yellow areas indicate suitable climate areas (EI = 10–20) and red areas indicate highly suitable climate areas (EI>20). Green dots indicate occurrence records of *L. camara*.

The current and potential distribution of lantana in Australia is shown in Figure 3. The occurrence records for Australia, which were reserved for model validation and not used for model fitting, accord well with the modelled climate suitability for the continent, and the present Australian distribution is consistent with the Ecoclimatic Index. Approximately 87% of the occurrence records fall within the suitable and highly suitable categories. In Australia, the model projects much of the eastern coast from Cape York in northern Queensland to southern New South Wales (NSW) to be climatically suitable (Figure 3). However, no occurrence records were found for Cape York Peninsula because despite a few isolated infestations in this region, lack of human disturbance limits the rate of spread [78]. Coastal areas in south-west Western Australia are projected to have suitable climate for lantana, conforming to the actual distribution since small infestations occur in these areas [1]. Central Australia is projected as being unsuitable, mainly due to dry stress.

### Future Climate

**Figure 4:**
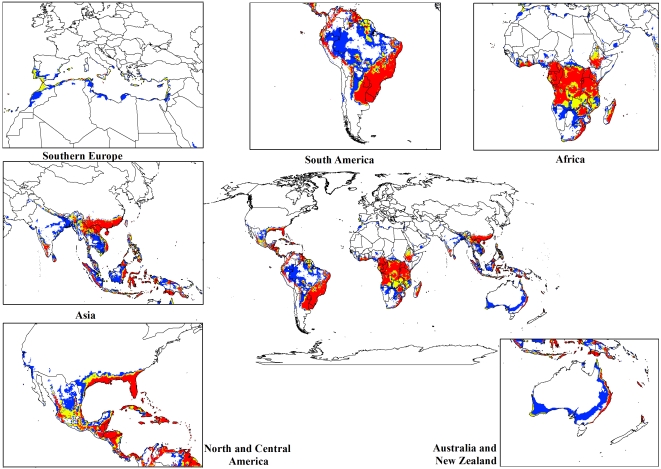
The climate (EI) for *L. camara* in the 2030s projected using CLIMEX under the CSIRO-Mk3.0 GCM running the SRES A1B scenario. White areas indicate unsuitable climate areas (EI = 0), blue areas indicate marginal climate areas (EI = 1–10), yellow areas indicate suitable climate areas (EI = 10–20) and red areas indicate highly suitable climate areas (EI>20).

**Figure 5:**
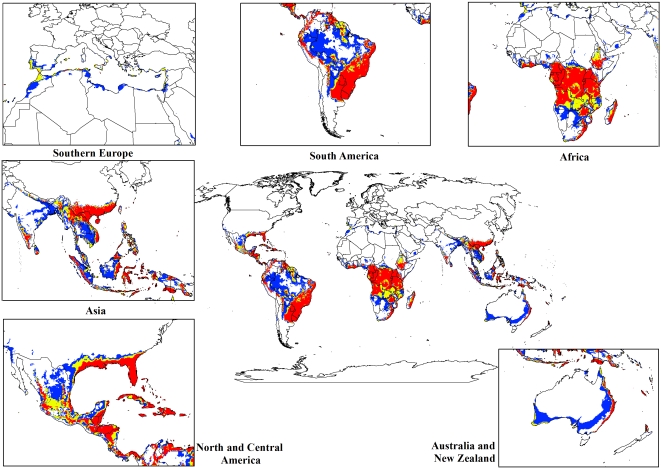
The climate (EI) for *L. camara* in the 2030s projected using CLIMEX under the CSIRO-Mk3.0 GCM running the SRES A2 scenario. White areas indicate unsuitable climate areas (EI = 0), blue areas indicate marginal climate areas (EI = 1–10), yellow areas indicate suitable climate areas (EI = 10–20) and red areas indicate highly suitable climate areas (EI>20).

**Figure 6:**
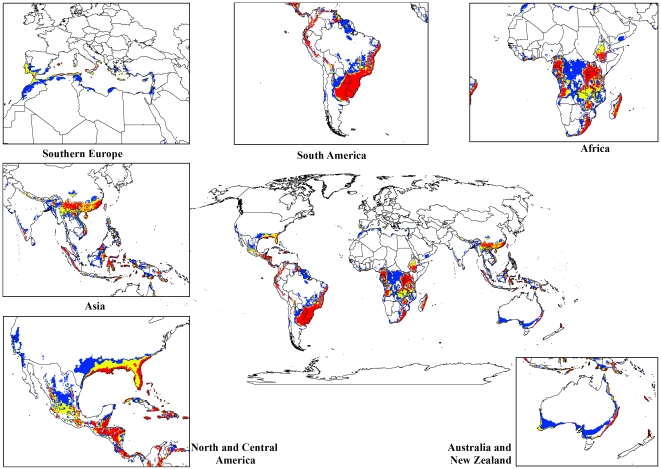
The climate (EI) for *L. camara* in the 2070s projected using CLIMEX under the CSIRO-Mk3.0 GCM running the SRES A1B scenario. White areas indicate unsuitable climate areas (EI = 0), blue areas indicate marginal climate areas (EI = 1–10), yellow areas indicate suitable climate areas (EI = 10–20) and red areas indicate highly suitable climate areas (EI>20).

**Figure 7:**
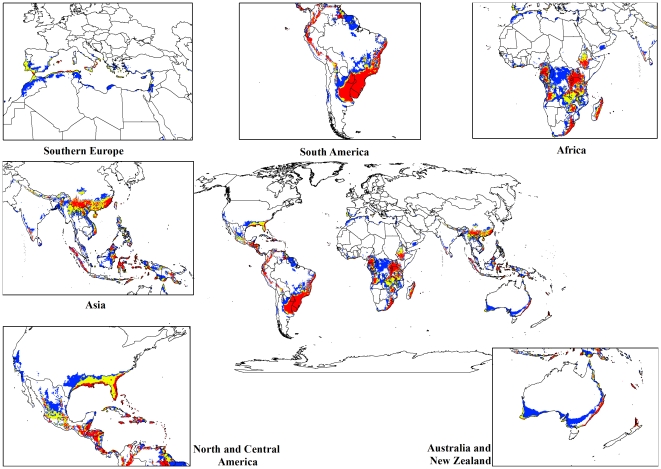
The climate (EI) for *L. camara* in the 2070s projected using CLIMEX under the CSIRO-Mk3.0 GCM running the SRES A2 scenario. White areas indicate unsuitable climate areas (EI = 0), blue areas indicate marginal climate areas (EI = 1–10), yellow areas indicate suitable climate areas (EI = 10–20) and red areas indicate highly suitable climate areas (EI>20).

**Figure 8:**
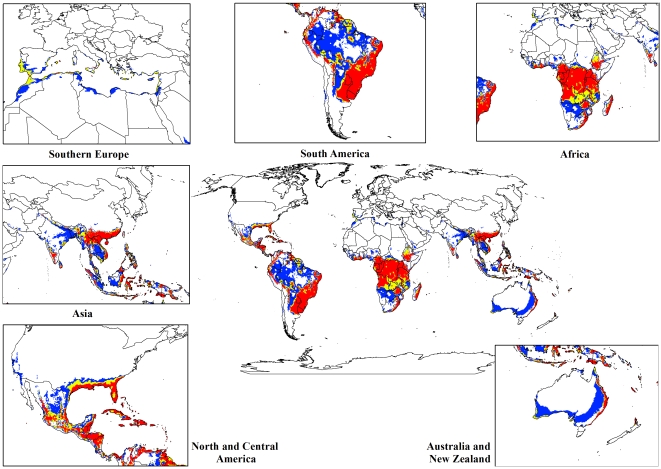
The climate (EI) for *L. camara* in the 2030s projected using CLIMEX under the MIROC-H GCM running the SRES A1B scenario. White areas indicate unsuitable climate areas (EI = 0), blue areas indicate marginal climate areas (EI = 1–10), yellow areas indicate suitable climate areas (EI = 10–20) and red areas indicate highly suitable climate areas (EI>20).

**Figure 9:**
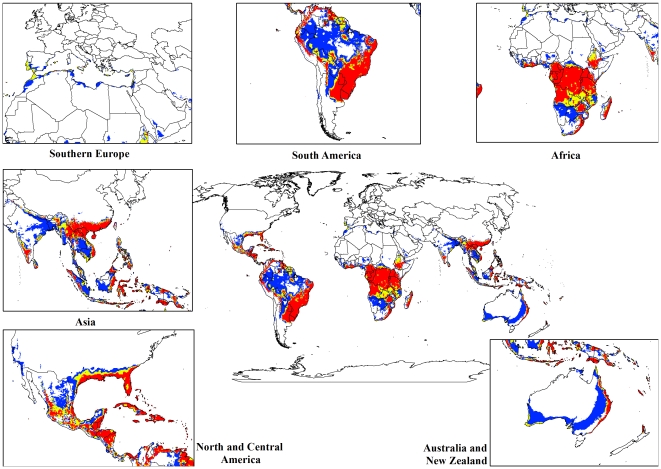
The climate (EI) for *L. camara* in the 2030s projected using CLIMEX under the MIROC-H GCM running the SRES A2 scenario. White areas indicate unsuitable climate areas (EI = 0), blue areas indicate marginal climate areas (EI = 1–10), yellow areas indicate suitable climate areas (EI = 10–20) and red areas indicate highly suitable climate areas (EI>20).

**Figure 10:**
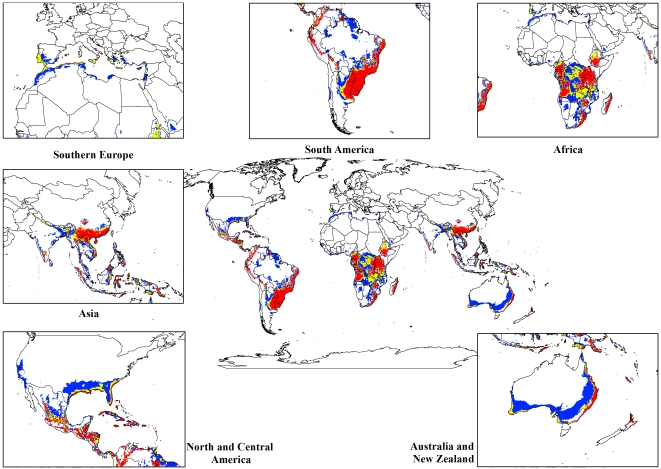
The climate (EI) for *L. camara* in the 2070s projected using CLIMEX under the MIROC-H GCM running the SRES A1B scenario. White areas indicate unsuitable climate areas (EI = 0), blue areas indicate marginal climate areas (EI = 1–10), yellow areas indicate suitable climate areas (EI = 10–20) and red areas indicate highly suitable climate areas (EI>20).

**Figure 11:**
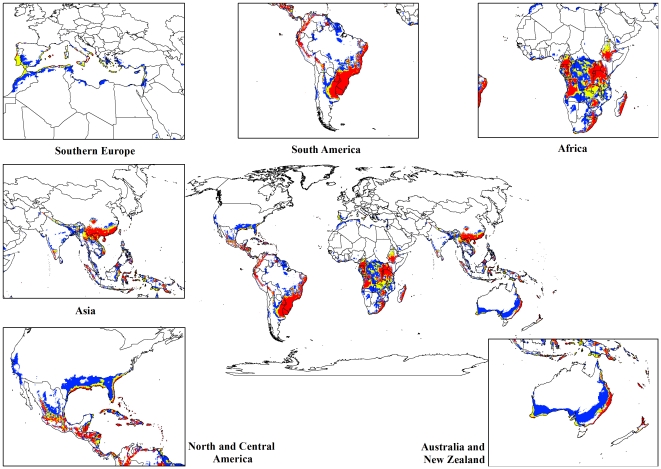
The climate (EI) for *L. camara* in the 2070s projected using CLIMEX under the MIROC-H GCM running the SRES A2 scenario. White areas indicate unsuitable climate areas (EI = 0), blue areas indicate marginal climate areas (EI = 1–10), yellow areas indicate suitable climate areas (EI = 10–20) and red areas indicate highly suitable climate areas (EI>20).

For both the climate change models, a contraction in the suitable climate areas was observed worldwide (Figures 4, 5, 6, 7, 8, 9, 10, and 11) with this trend exacerbated in the 2070 scenario. The two GCMs showed moderately variable results but within each of the models, minimal sensitivity was seen between the two emission scenarios.

In South America, suitable climate areas for lantana are substantially reduced throughout northern Argentina, Uruguay, Bolivia, Peru, Paraguay, large parts of Brazil, French Guiana, Surinam, Guyana, coastal Venezuela and Colombia. A similar trend is seen in Central America with suitable climate areas for lantana contracting in Panama, Costa Rica, Nicaragua, Honduras and Guatemala. By 2030, a reduction in suitable climate for lantana is projected in all of these countries and this trend is exacerbated by 2070 (Figures 6, 7, 10, and 11). Warming under future climate scenarios is projected to lead to a substantial reduction in suitable climate for lantana in this region. In North America, some differences can be seen between the two GCMs in coastal areas of southern states such as Florida, Louisiana and Texas in North America. Under the CSIRO-Mk3.0 GCM, these areas are projected to remain climatically suitable until 2070 (Figures 6 and 7) while the same areas are projected as marginal to unsuitable with the MIROC-H GCM (Figures 10 and 11).

In Africa, suitable climatic areas for lantana are projected to contract substantially with only parts of Ethiopia, Uganda, Tanzania, Zambia, Angola, Gabon and Republic of Congo remaining suitable in 2070 under both GCMs and both SRES scenarios (Figures 6, 7, 10, and 11). Nevertheless, much of the continent shows high climatic suitability for lantana until 2030 (Figures 4, 5, 8, and 9). In South Africa, lantana range appears to expand further inland, mainly in the Eastern Cape and Kwazulu-Natal provinces, west of the Swaziland border as well as into Lesotho and this is particularly apparent by 2070 under both GCMs.

In Asia, there is a considerable reduction in the projected potential range under climate change scenarios, especially for countries such as India, Sri Lanka, Myanmar, Thailand, Cambodia and Vietnam. However, in China the potential range shifts further inland and this is especially noticeable in the MIROC-H 2070 scenario (Figures 10 and 11). Lantana potential range shifts south into new areas in Australia (Victoria, South Australia and Tasmania) and a range expansion is seen in the south-west corner of Western Australia under both GCMs. Coastal areas in North Africa (Morocco and Algeria) and Southern Europe (Portugal, Spain, Italy and Greece) are projected to have suitable climate areas for lantana by 2070, particularly under the CSIRO-Mk3.0 GCM (Figures 6 and 7).

## Discussion

This study has modelled the suitable climate area for *Lantana camara* under current climate and future climate scenarios using CLIMEX. The model provides a good fit to the current global distribution records as well as the current Australian distribution, which was reserved for model validation purposes. Under historical climate, much of the tropics and subtropics are modelled as having suitable climatic conditions for lantana. On the African continent, most of eastern and central Africa, parts of West Africa as well as eastern Madagascar are projected to have suitable to highly suitable climatic conditions for lantana greatly exceeding its current known distribution here. This could be a function of the lack of reporting from this region or invasion lag. Other non-climatic factors such as lack of dispersal opportunities or biotic interactions could also inhibit lantana from spreading in these regions. These results highlight areas where more detailed risk assessments on lantana invasion may be prudent. Capacity building, effective implementation of existing laws on movement and transport of lantana together with a public education campaign in this region may contribute towards more effective management. A similar case could be made for Asia where lantana's potential distribution exceeds its current distribution. Maps such as the ones produced in this study can be a useful tool in public awareness campaigns so as to enlist the help of local communities in the management of existing infestations and the prevention of further invasion.

Although cold stress appears to be the main factor limiting its distribution, dry stress prevents its establishment in the drier western parts of South Africa and inland Australia. Within the marginal areas identified in inland Australia, lantana would be patchily distributed and restricted to favourable microhabitats. Thus in these areas, it would pose limited threat and dispersal is also likely to be very slow in these regions.

The results of the climate change modelling give an indication of the possible changes in the potential distribution of lantana. As the climate changes, some areas where lantana currently occurs may become climatically unsuitable. All scenarios considered in this study indicated an overall contraction in the climatically suitable area for lantana in the future (Figures 4, 5, 6, 7, 8, 9, 10, and 11). Some of this reduced potential range for lantana covers important biodiversity hotspots of the world (e. g., coastal forests of Eastern Africa, lowland forests of west Africa, Indo-Burma region, Western Ghats of India and Sri Lanka). Whilst this result is likely to be encouraging for weed managers, it is probably paralleled by similar climatic threats to biodiversity [79]. Nonetheless, these results may be useful in making informed choices about the allocation of resources for weed control by highlighting areas where climate suitability is expected to decrease in the future.

The results identify new areas of the world that may be at risk of lantana invasion due to changes in climate, and which may warrant strategic control measures to prevent its spread. Although an overall reduction in the potential distribution is projected in the Americas, Africa and Asia under the future climate scenarios examined here, the potential for range expansion in North Africa, Europe, Australia and New Zealand was identified. In South Africa and China, lantana's potential distribution may expand further inland into new areas in the future. Such areas may require more detailed risk assessments on lantana invasion. The assessment and management of risks from weeds depends, to a large extent, on projections of habitat suitability so that threat levels can be assessed. The response of exotic species to changes in climate must form an integral part of such assessments [80], [81]. In particular, areas that are currently at risk and that will continue to be at risk from lantana in the future are identified in this study. These areas could be important for biodiversity conservation, particularly in biodiversity hotspots such as southwest Australia, Atlantic Forests of South America and Caribbean Islands. These areas also include important agricultural areas worldwide such as southern states of North America, coastal areas of southern China, east coast of Australia and South Africa. Our results can be used in decision-making processes by land managers in prioritizing areas for eradication and in determining areas where containment would be cost-effective [82].

Under future climates, lantana may expand into areas that are currently too cool for it to survive and this can be seen in improved suitability in Europe. Biosecurity agencies in these countries should be aware of this potential threat and monitor areas that have been identified in this study for early signs of outlying lantana populations becoming invasive. Currently lantana is grown as an ornamental potted plant in northern Italy and is widely used in private and public gardens in central and southern Italy [83]. Areas of Portugal and Greece have also been identified as becoming climatically suitable for lantana to naturalize in the future. Simple and low-cost strategies such as weed alerts, identification and distribution of replacement garden ornamentals, low-cost surveillance and hygiene efforts to prevent lantana spreading to new areas may be a worthwhile investment on the part of biosecurity agencies in these countries. Climatic suitability for lantana may decrease, even leading to range contraction, in places where conditions become too warm and wet (e.g. northern Australia). Changes in climate may also have implications for the biological control of lantana since the distribution of biocontrol agents will also likely alter with climate change [84]. Lantana was one of the first weeds to be targeted for classical biological control at the turn of the century [53]. Since then 36 insect species have been released in 33 countries throughout its invaded range with disappointing results [48]. It will be important to establish ongoing monitoring of current biological control programs for lantana throughout its invaded range so that changes may be detected early and appropriate action taken.

Since CLIMEX is based on climate, non-climatic factors that affect species' distributions such as dispersal potential, biotic interactions and type of habitat are not included explicitly in the modelling process. However, the modelling method employed here should capture any effects from the release from natural enemies [70] that are apparent in lantana's exotic range, thus approximating its fundamental niche [14]. Moreover, the uncertainties associated with the state of climate modelling and uncertainty in future global greenhouse gas emission patterns [85] mean that models based on future climate scenarios should be treated as elaborate sensitivity analyses, indicative of the direction and magnitude of change that may be expected in the future. The climate suitability *projections* show areas of climatic suitability for lantana and are not *predicted* future distributions. Lantana's bird-dispersed berries make it an effective disperser [86], able to expand its range rapidly to occupy a broad range of environments within its climatic tolerance. Once established, lantana can survive long periods of drought [30], [86]. It can also grow on poor soils and pure sand substrates if there is adequate soil moisture [63], [75].

The climate models for lantana presented here may be useful for its management, particularly under future climate change. These models may be adapted for: informing decisions regarding allocation of resources for weed management towards areas where risk of invasion remains and away from areas where climatic suitability is likely to decrease under future climate, inform management decisions in preventing the spread of lantana into new areas, and prioritizing lantana management initiatives in areas which are currently at risk and will remain at risk of invasion in the future.

The modelling presented here ignored the direct effects of increasing concentrations of atmospheric CO_2_ on factors that affect the ability of lantana to grow and persist (e.g. its water use efficiency). It would be useful to supplement our knowledge of the direct effects of increasing [CO_2_] on invasive plants in general at both a physiological level and at the ecosystem level. Because of the expense and difficulties of running free-air carbon emission (FACE) studies, there have been very few studies of ecosystem water use efficiency. The differences in projected range changes based on assumptions drawn from studies of individual plants [37] are markedly different to those based on results from FACE experiments [37], [87]. Cheaper “open top" CO_2_ experiments may offer a compromise that would allow researchers to explore single plant and ecosystem water use efficiency changes for a broad range of species and climate types. This knowledge could have profound impacts on our ability to model likely range changes under future climates.

Those areas that have been identified as suitable or highly suitable for lantana are at greatest risk, and the projection of future climate scenarios provides useful insights into the potential distribution of this highly invasive weed. The identification of important biodiversity conservation and agricultural assets that may be affected by the anticipated changes in range of this species as well as new areas at risk of invasion under climate change should facilitate strategies to manage its spread.
